# A Fisher Information Theory of Aesthetic Preference for Complexity

**DOI:** 10.3390/e26110901

**Published:** 2024-10-24

**Authors:** Sébastien Berquet, Hassan Aleem, Norberto M. Grzywacz

**Affiliations:** 1 Department of Biology, Loyola University Chicago, Chicago, IL 60660, USA; sberque1@jh.edu; 2 Department of Biomedical Engineering, Johns Hopkins University, Baltimore, MD 21218, USA; 3 Department of Molecular Pharmacology and Neuroscience, Loyola University Chicago, Chicago, IL 60660, USA; ha438@georgetown.edu; 4 Departments of Psychology, Loyola University Chicago, Chicago, IL 60660, USA; 5 Department of Cognitive Science, Johns Hopkins University, Baltimore, MD 21218, USA

**Keywords:** aesthetic preference for complexity, inverted U-Shape behavior, observed Fisher information, statistics of image complexities, urban and natural environments

## Abstract

When evaluating sensory stimuli, people tend to prefer those with not too little or not too much complexity. A recent theoretical proposal for this phenomenon is that preference has a direct link to the Observed Fisher Information that a stimulus carries about the environment. To make this theory complete, one must specify the model that the brain has about complexities in the world. Here, we develop this model by first obtaining the distributions of three indices of complexity measured as normalized Shannon Entropy in real-world images from seven environments. We then search for a parametric model that accounts for these distributions. Finally, we measure the Observed Fisher Information that each image has about the parameters of this model. The results show that with few exceptions, the distributions of image complexities are unimodal, have negative skewness, and are leptokurtotic. Moreover, the sign and magnitude of the skewness varies systematically with the location of the mode. After investigating tens of models for these distributions, we show that the Logit-Losev function, a generalization of the hyperbolic-secant distribution, fits them well. The Observed Fisher Information for this model shows the inverted-U-shape behavior of complexity preference. Finally, we discuss ways to test our Fisher-Information theory.

## 1. Introduction

Decision-making in all corners of our lives requires information [1,2,3,4]. Evidence that the brain likes large amounts of information includes a dislike of too little complexity in sensory signals [5,6,7] and addiction to tools designed to provide a lot of data to our senses [8,9,10]. However, the brain has limited resources, and thus, it cannot deal with too much information [11,12,13]. The brain, therefore, dislikes incoming sensory signals with too much complexity [14,15,16]. Thus, one observes a ubiquitous relationship between complexity and preference for sensory signals, that is, an inverted-U shape behavior [15,17,18]. This behavior has been used to suggest that the brain continuously measures and monitors the amount of incoming sensory information [4]. The brain may then use this amount to decide how many resources to devote to the processing of the information [4,19,20,21]. Thus, this amount may be a metacognitive signal related to aesthetic preference, like, for example, processing fluency [22].

Different hypotheses try to explain the inverted-U-shape behavior as a function of complexity or amount of information [15,23,24]. A simple hypothesis is that people like sensory stimuli with the most common amounts of information in the surrounding environment. However, people do not necessarily always prefer the most common complexities [25]. In an alternate hypothesis, too little information has been called boring [23,24,26] and too much information has been called confusing [23,27,28]. However, none of these hypotheses explain how the brain knows what is too little or too much. A recent theoretical proposal has tried to overcome this limitation [4]. This theory begins with the observation that as the environment changes (for example, through time or movement), the statistical properties of the stimuli also vary. Imagine that the brain has a general parametric model of these properties across environments, except that as they vary, the best parameters do, too [22,29,30,31]. Thus, the brain should like stimuli that are especially informative about the parameters. The suitability of a stimulus to inform about the parameters is best measured with Observed Fisher Information [32,33]. The new proposal then suggests that stimuli eliciting the most liked amount of information are those yielding the most Observed Fisher Information about the parameters [4]. The authors of this proposal have suggested that it may account for the inverted U-shape relationship between aesthetic pleasure and stimulus complexity.

If this Observed-Fisher-Information theory is correct, one must have a good guess of the model that the brain may use to measure the amounts of information in sensory stimuli. One way to answer this question is to study them in the world itself. For vision, for example, one may measure the distributions of image complexities from different environments [34,35,36]. From these measurements, one may try to reverse-engineer what models may capture these statistical distributions. Such a statistical approach has been used successfully in the study of sensory systems of the brain. For example, models generated from such statistical studies of natural images have been successful in accounting for various aspects of the organization of the brain’s visual system [37,38,39,40,41].

In this article, we test the possibility that Observed Fisher Information could be a useful metric for aesthetic values related to amounts of information in visual stimuli. To perform this test, we first measure visual complexity in hundreds of natural and human-made scenes across seven environments. In this article, we focus on three types of complexity, namely, luminance, chromatic, and spatial. Luminance complexity refers to the flatness of the distribution of intensities across the pixels of an image, as measured by Shannon Entropy. Similarly, chromatic complexity refers to the flatness of the distribution of hues in the same pixels. Finally, spatial complexity refers to a two-dimensional distribution of intensities, that is, considering the correlation between pixels in two locations. Then, we design a general statistical model that captures the distribution of complexities across environments. This model allows us to measure Observed Fisher Information for individual images. Using this model, we test whether the Observed Fisher Information for images in any environment shows an inverted-U-shape relationship with complexity.

## 2. Theory

### 2.1. Preliminaries

**Figure 1:**
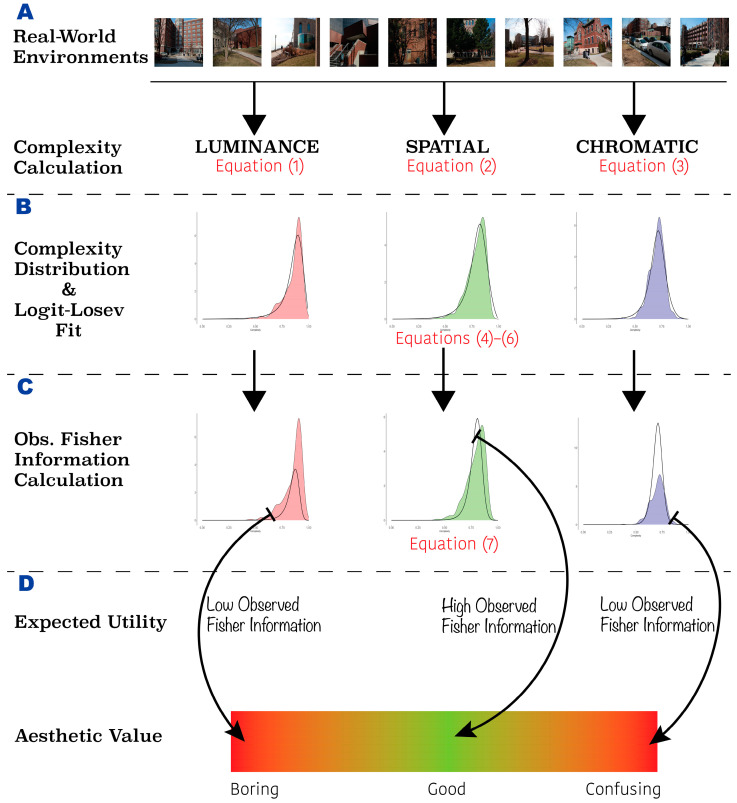
Theoretical framework for the connection between the amount of information and aesthetic values. (**A**) Images can come from multiple environments in the world. Several types of complexities, that is, amounts of information, are computed from each image. (**B**) The probability distribution for each type of complexity and environment is built. A model of the likelihood function is fit to the distribution to find the best parameters. (**C**) The Observed Fisher Information curve is computed from the model. (**D**) Only the complexities yielding the largest observed fisher information have high utility and thus good aesthetic value. Too little complexity creates a boring image, while too much complexity creates a confusing image.

The goal of this article is to find a suitable model for the distribution of complexities across environments. This model will underpin the Observed-Fisher-Information theoretical framework of aesthetic preference for complexity. We then assess whether the Observed Fisher Information calculated from this model captures the inverted-U-shape preference curve seen as complexity varies. To achieve this goal, the work in this article proceeds in three steps (Figure 1):

- In the first step, we compute three measures of the amount of information in images from seven natural or human-made environments (Figure 1A). These measures use Shannon Entropy, whose normalized versions are complexities [25,42]. In this article, we measure complexities based on luminance, spatial, and chromatic information. Therefore, because we have many images per environment, we can build theories for each of the three estimated distributions (histograms or kernel-density distributions) of the distributions of complexities.
- We then fit analytical models of probability density functions to these estimated distributions (Figure 1B). The goal is to find a single model that can fit all twenty-one of them (three measures of complexity and seven environments) by simply selecting the right parameters. We make sure that the models are simple, having at most two parameters.
- Finally, we calculate the Observed Fisher Information for each of the three complexities obtained for each image (Figure 1C). This measure supplies the amount of information that the image has about the parameters of the model. Such a measure is important. Without it, we cannot be sure what the best model parameters are for the image that we currently see, because environments are constantly changing. We also calculate in this article the expected Observed Fisher Information for each environment and complexity type. This expectation is known as the Fisher Information, providing a measure of how easily the environment can be understood.

### 2.2. Amount of Information

The first of our theoretical steps is to define complexity (Figure 1A). In some past studies, visual complexity was defined in simple terms, such as the number of features or a perceptual scale [14,17,43,44]. In this study, we follow other studies that wanted to define complexity more rigorously and in a way that would be consistent across studies [4,25]. This is to define complexity as a normalized amount of information [25]. Here, we begin with luminance complexity. For each Image Q, we first convert it to grayscale (the rec601 luma component) and then obtain the probability $P_{Q}^{\left(L\right)}\left(I\right)$ of Intensity I. Then, using this probability and calculating the normalized expected Shannon entropy, we obtain the luminance complexity

(1) $$c_{L}\left(Q\right)=-\sum_{I=0}^{I^{*}}{P_{Q}^{\left(L\right)}\left(I\right)log}_{I^{*}+1}\left(P_{Q}^{\left(L\right)}\left(I\right)\right)\;\;,$$

where $I^{*}$ is the maximally possible intensity (255 in this article). In this equation, $I^{*}+1$ is the base of the logarithm, which is not in a natural base because of the normalization [25]. The normalization in luminance complexity is such that ${0\leq c}_{L}\left(Q\right)\leq 1$. This index of complexity is zero only for single-tone images (the simplest ones) and $c_{L}\left(Q\right)=1$ for images whose intensities spread homogeneously and randomly through all values.

In turn, spatial complexity considers the amount of information due to both intensity and spatial organization. We generalize the procedure used for Equation (1). This generalization first measures the probability $P_{Q}^{\left(S\right)}\left(I_{2}|I_{1},T\right)$ in image Q that a pixel with intensity $I_{1}$ is juxtaposed with a pixel with intensity $I_{2}$ after the isometric transformation T. From this measurement, we define spatial complexity following the same steps as for Equation (1) to obtain

(2) $$c_{S}\left(Q,T\right)=-\sum_{I_{1}=0}^{I^{*}}P_{Q}^{\left(L\right)}\left(I_{1}\right)\sum_{l_{2}=0}^{I^{*}}P_{Q}^{\left(S\right)}\left(I_{2}|I_{1},T\right){log}_{I^{*}+1}\left(P_{Q}^{\left(S\right)}\left(I_{2}|I_{1},T\right)\right)\;.$$

Again, because of the normalization, $0\leq c_{S}\left(Q,T\right)\leq 1$. In the figures reporting spatial complexity throughout the article, we follow the conventions that we reported elsewhere and plot the mean of the overall possible transformation T [25].

Finally, we compute chromatic complexity, which we define here for the first time. For each image Q, we first convert it from RGB to HSV and extract the probability $P_{Q}^{\left(C\right)}\left(h\right)$ of hue h. We then generalize Equation (1) to obtain

(3) $$c_{C}\left(Q\right)=-\sum_{h=1}^{h^{*}}{P_{Q}^{\left(C\right)}\left(h\right)log}_{I^{*}}\left(P_{Q}^{\left(C\right)}\left(h\right)\right)\;\;,$$

where $h^{*}$ is the number of possible hues. Once more, $0\leq C\left(Q\right)\leq 1$.

### 2.3. Likelihood Models

The second step of our theoretical work is to find good analytical models of probability density functions to fit the distributions of complexities (Figure 1B). We make sure that the models are simple, having at most two parameters. We searched for these models broadly, considering mathematically or computationally tens of different continuous, finite-support probability distributions [45,46]. Among these distributions, the ones that came closest were the Beta, Logit-Normal, and Logit-Losev distributions. We describe them in this section.

The probability density function of a Beta distribution of complexities is

(4) $$P_{B}\left(c_{x}|\alpha ,\beta \right)=\frac{c_{x}^{\alpha -1}{\left(1-c_{x}\right)}^{\beta -1}}{B\left(\alpha ,\beta \right)}\;,$$

where $c_{x}$ is one of the three complexity types described in Equations (1)–(3), α>0 and β>0 are the parameters, and

$$B\left(\alpha ,\beta \right)=\frac{\Gamma \left(\alpha \right)\Gamma \left(\beta \right)}{\Gamma \left(\alpha +\beta \right)}\;,$$

where Γ is the gamma function. Next, the Logit-Normal distribution of complexities is

(5) $$P_{N}\left(c_{x}|\mu_{N},\sigma \right)=\frac{1}{\sqrt{2\pi }\sigma c_{x}\left(1-c_{x}\right)}e^{-\frac{{\left(\mathrm{logit}\left(c_{x}\right)-\mu_{N}\right)}^{2}}{2\sigma^{2}}},\;$$

where $\mu_{N}$ and σ are the parameters, and

$$\mathrm{logit}\left(c_{x}\right)=\ln\left(\frac{c_{x}}{1-c_{x}}\right)\;.$$

Finally, the Logit-Losev probability density function is a modification of a distribution studied by Losev [47], itself a generalization of the hyperbolic-secant distribution [48]. The modification is the transformation of the independent variable with the logit function, making the outcome a finite-support distribution. This distribution is introduced in this article for the first time. The general form of this distribution is

$$P_{L}\left(c_{x}|\mu ,a,b\right)=\frac{N\left(a,b\right)}{c_{x}\left(1-c_{x}\right)\left(e^{-a\left(\mathrm{logit}\left(c_{x}\right)-\mu \right)}+e^{b\left(\mathrm{logit}\left(c_{x}\right)-\mu \right)}\right)}\;,$$

where μ, a>0, and b>0 are the parameters, and $N\left(a,b\right)$ is the normalization constant. Although this function has three parameters, we use here a simplified version with only two parameters by making a=b, that is,

(6) $$P_{L}\left(c_{x}|\mu ,a\right)=\frac{N\left(a\right)}{c_{x}\left(1-c_{x}\right)\left(e^{-a\left(\mathrm{logit}\left(c_{x}\right)-\mu \right)}+e^{a\left(\mathrm{logit}\left(c_{x}\right)-\mu \right)}\right)}\;,$$

where the normalization constant is $N\left(a\right)=2a/\pi$.

### 2.4. Fisher Information

The last step of our theoretical work involves the computations of Observed Fisher Information (Figure 1C). These computations use Equations (4)–(6), which define the probability density distributions of complexities, $P_{y}\left(c_{x}|\theta_{1},\theta_{2}\right)$, where $\theta_{1}$ and $\theta_{2}$ are the two parameters. Then, the log-likelihood of the parameters $\theta_{1}$ and $\theta_{2}$ given the data $c_{x}$ is

$$l_{y}\left(\theta_{1},\theta_{2}|c_{x}\right)=\ln\left(P_{y}\left(c_{x}|\theta_{1},\theta_{2}\right)\right)\;.$$

The Observed Fisher Information matrix at $\theta_{1}^{*}$ and $\theta_{2}^{*}$ is

(7) $$F_{y}\left(\theta_{1}^{*},\theta_{2}^{*},c_{x}\right)=-{\left.\left(\begin{array}{cc} \frac{\partial^{2}}{\partial \theta_{1}^{2}} & \frac{\partial^{2}}{\partial \theta_{1}\partial \theta_{2}}\\ \frac{\partial^{2}}{\partial \theta_{2}\partial \theta_{1}} & \frac{\partial^{2}}{\partial \theta_{2}^{2}} \end{array}\right)l_{y}\left(\theta_{1},\theta_{2}|c_{x}\right)\right|}_{\left(\theta_{1},\theta_{2}\right)=\left(\theta_{1}^{*},\theta_{2}^{*}\right)}\;,$$

where the symbol ∂ marks partial differential equations of the log-likelihood by its parameters. In our case, one can interpret this equation as follows: If the current parameters of the internal model for estimating the likelihood of an image with complexity $c_{x}$ are $\left(\theta_{1}^{*},\theta_{2}^{*}\right)$, then the Observed Fisher Information is $F_{y}$. The diagonal components of $F_{y}$ are the curvature of the graph of the log-likelihood. Near the maximum-likelihood estimate, low Observed Fisher information shows that the maximum is “blunt”. Conversely, high Observed Fisher Information shows that the maximum is sharp. The off-diagonal components of $F_{y}$ show the co-dependence between the parameters.

The application of Equation (7) to Equations (4)–(6) is important to understand the results of this article. For example, the first diagonal element for the Beta distribution (that for α) gives

(8) $$F_{B,1,1}\left(\alpha ,\beta ,c_{x}\right)=\psi_{1}\left(\alpha \right)-\psi_{1}\left(\alpha +\beta \right)\;,$$

where $\psi_{1}$ is the trigamma function. As we will discuss later, the importance of this result is that it is independent of $c_{x}$. A similar independence appears for the first diagonal element for the Logit-Normal distribution (that for $\mu_{N}$):

(9) $$F_{N,1,1}\left(\mu_{N},\sigma ,c_{x}\right)=\frac{1}{\sigma^{2}}\;.$$

However, when using the Logit-Losev distribution (Equation (6)), the first diagonal element (that for μ) is dependent on $c_{x}$:

(10) $$F_{L,1,1}\left(\mu ,a,c_{x}\right)=\frac{4a^{2}}{{\left(e^{-a\left(\mathrm{logit}\left(c_{x}\right)-\mu \right)}+e^{a\left(\mathrm{logit}\left(c_{x}\right)-\mu \right)}\right)}^{2}}\;.$$

As we will discuss later, this function shows an inverted-U-shape behavior as a function of $c_{x}$. This behavior is such that the optimal $c_{x}$ is

(11) $$c_{x}^{\left(opt\right)}=\frac{1}{1+e^{-\mu }}$$

and at this complexity, the Observed Fisher Information is

(12) $$F_{L,1,1}\left(\mu ,a,c_{x}^{\left(opt\right)}\right)=a^{2}\;.$$

From Observed Fisher Information in Equation (7), we can also compute the full Fisher Information matrix [49,50]. This is the expectation of the Observed Fisher Information matrix:

(13) $$F_{L,1,1}\left(\mu ,a,c_{x}^{\left(opt\right)}\right)=a^{2}\;.$$

## 3. Materials and Methods

### 3.1. Photography

We photographed 1000 images of natural and human-made environments randomly. Because people are not very good at doing things randomly [51], we tried to orchestrate this by photographing with a camera in-hand and without looking at the scene to frame it. We did so to prevent the photographer from imposing their aesthetic biases on the images. Without these biases, the distribution of complexities would be faithful to the signals from the external world, not to the mind of the photographer. This would allow us to test whether this distribution had a universal shape. If so, the brain could use this shape as a likelihood distribution.

The camera was always placed in ‘landscape mode’ (horizontal pictures) and its orientation varied from about −30° below the horizon (aimed towards the ground in front of the photographer) to about +30° above it. Thus, we avoided capturing too much of the sky. Finally, the height of the camera was at the human-eye level. Although we photographed the images without framing, none of them were blurry or had overlapping parts after inspection. The images fell into seven distinct environments based on location. These environments were Parks (157 images), College Campuses (239 images), Small Streets (185 images), Large Streets (180 images), Snowy Rural Settings (70 images), Malls (94 images), and Forests (75 images). We chose these environments to make sure that we obtain a high diversity of surroundings, from urban to rural to natural. Moreover, we obtained images from a high variety of urban environments. All these images are available in the supplementary information at https://osf.io/23auc (accessed on 14 November 2023).

The images were taken in two groups. The first was of pictures from Parks, Campuses, and Street environments. We obtained these pictures with a Canon EOS 5D Mark II camera (5616 × 3744 pixels) and a Canon Zoom Lens (EF 30 mm). All images were taken using a manual setting to prevent automatic resetting by the camera from affecting our statistics. The only setting that varied between environments was the camera’s shutter speed (1/500 s to 1/2500 s). This change was necessary because different environments elicited dissimilar light exposures. Therefore, if we kept a constant shutter speed, the quality of the images would favor some environments over others in terms of image quality. We kept the ISO constant (ISO = 200) across all environments and fixed the focal for each environment (f4.0–f5.0). We captured the Campus environment at around 4 pm on 21 March 2022 at the Lakeshore Campus of Loyola University Chicago. In turn, we captured the Small Street environment in the Gold Coast neighborhood of Chicago at around 5 pm on 16 March 2022. The Large Street and Parks environments were captured between 4 and 6 pm on 15 June 2022 in downtown Chicago for the former and in Lincoln Park for the latter. The second group of images, namely, in Malls, Farmland and Forests were all obtained in analogous manner (same photographic settings) in the suburbs of Chicago between 9 and 11 am on 7 April 2022. We obtained these images using an Apeman A80 1080 p HD camera (5120 × 3640 pixels).

A concern was whether image noise could affect our measurements of complexity. However, we estimated the effect of noise on the various complexities to be so small that we could neglect it. The estimation used measurements in the literature showing that if the noise was imperceptible in an image, the signal-to-noise ratio was 40 dB or more [52], that is, more than 100:1.

### 3.2. Quantitative Analysis

After separating the images into environments, we used a MATLAB (Mathworks, Natick, MA, USA) code written in-house to obtain the three complexity types for each image (Equations (1)–(3)—Figure 1A). However, in our analysis of the Snowy Rural environment, we could use only 55 of the 70 images for spatial complexity measurements. The limitation in this environment was that sometimes there was not enough spatial information available due to the snow. We then obtained the kernel-density distribution for each environment with the geom_density function from the R-Studio ggplot Package [53]. Next, we used Hartigan’s Dip-Test [54] to evaluate whether the distributions of complexities were unimodal. We had two reasons to apply this test: First, we wanted to make sure that the models were simple. Therefore, we needed to know how complex we needed to build it. In this regard, we felt that multimodality was a possibility for the model, and if so, it would have to be more complex. Therefore, we assessed this possibility, using the best available probe, namely, the Hartigan’s Dip test. Second, one cannot always visually assess the number of modes in a sampled distribution. Even unimodal distributions can show a multimodal density in their sampled representation, particularly when one uses samples limited in size. Afterward, we obtained four descriptive statistics from the distribution by using various R packages. We began with the median and median absolute deviation (MAD). We then obtained the skewness and used the D’Agostino K^2^ test [55] to probe whether this statistic was significantly different from zero. Finally, we measured excess kurtosis and whether it was significantly different from zero by using Bootstrapping [56].

We then searched for good, analytic likelihood-function models to fit our distributions of complexities, as described in Section 2 (Figure 1B). In this search, we used a MATLAB code written in-house to fit the distributions, employing as a metric the $\chi^{2}$ distance between the distributions and the models. We probed the significance of the fits with $\chi^{2}$ tests.

Finally, we used these fitted analytic likelihood-function models to calculate the Observed Fisher Information for each environment and complexity type (Equation (7)—Figure 1C). These calculations also used a MATLAB code written in-house. From the results of these calculations, we obtained overall Fisher Information for each condition by computing the expected Observed Information (Equation (11)).

## 4. Results

### 4.1. Distribution of Complexities in Natural and Human-Made Environments

**Figure 2:**
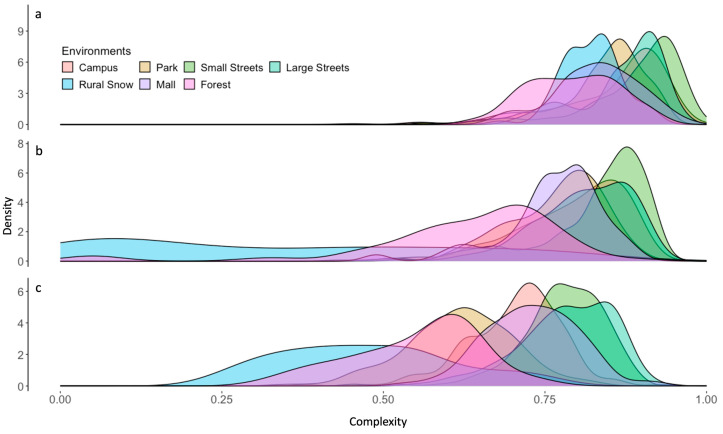
Probability density distribution for seven environments (color-coded) and three types of complexity. (**a**) Luminance complexity. (**b**) Spatial complexity. (**c**) Chromatic complexity. The distributions of complexities are different across complexity types in terms of magnitudes, spread, overlap, and order of peak complexities.

**Figure 3:**
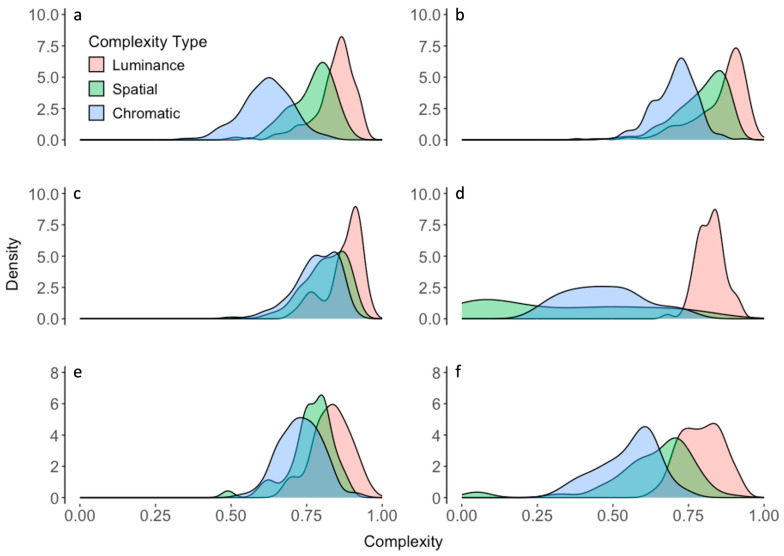
Probability density distribution for three types of complexity (color-coded) and six environments. (**a**) Parks. (**b**) College campus. (**c**) Large streets. (**d**) Snowy rural settings. (**e**) Malls. (**f**) Forests. Our seventh environment, namely, small streets, exhibits properties that are like those in Panel (**c**). The distributions of complexities are different across environments in terms of magnitudes, spread, overlap, and order of peak complexities.

The amount of information conveyed by a sensory stimulus appears to underlie an aesthetic experience. However, to be aesthetic, this amount must be right, neither too much nor too little. Thus, one sees a ubiquitous relationship between aesthetic preference and sensory complexity (the latter being the proxy for the amount of information). This relationship is commonly termed the inverted-U-shape behavior [15,17,18]. We have hypothesized that this behavior stems from aesthetic pleasure following the Observed Fisher Information conveyed by a sensory stimulus [4]. The goal of this article is to provide a first test of this hypothesis by first building a good likelihood-function model of how the brain estimates the probability of complexities in the world and then using this model to estimate Observed Fisher Information. To be most useful to the brain, this model should capture the statistics of complexities in the real world. We have thus measured the distribution of visual complexities across seven different environments. In addition, we used three types of complexity, namely, luminance, spatial, and chromatic complexities. The distributions for the three types of complexities and seven environments appear in Figure 2 and Figure 3.

Figure 2 shows that each environment can be differentiated from the others based on its unique set of complexity distributions. Nevertheless, the distributions in Figure 2 appear to have four important “universal” properties that any model must obey: First, the distributions across environments are unimodal, that is, except for occasional noise fluctuations, the curves only have a single peak complexity. This unimodality is confirmed statistically with the Hartigan’s Dip Test. Second, no peak complexity falls below 0.5, aside from the spatial and chromatic complexities of the snowy rural environment. Third, the distributions tend to show skewness set by the position of the peak, such that when it is above 0.5, the skewness is negative and vice versa. Fourth, the distributions tend to show positive excess kurtosis, that is, they are leptokurtic. Hence, their peaks are narrow (high curvature), and their tails are fat (long). The only significant exception is the spatial complexity of snowy rural images, whose distribution is platykurtic. Another significant feature of Figure 2 is that complexities vary systematically with the environment. For example, forests, parks, and snowy rural settings tend to have the lowest complexities. In contrast, they are highest in urban streets. All the statistics confirming these trends of the distributions of complexity are available in the supplementary information at https://osf.io/23auc (accessed on 14 November 2023).

Figure 3 shows that the snowy rural environment is somewhat of an outlier. It is the only environment whose distribution of spatial complexities peaks near zero. In addition, such a near-zero peak causes the negative excess kurtosis of this distribution (supplementary information, https://osf.io/23auc, accessed on 14 November 2023). This environment also shows the most spread of density distributions for both spatial and chromatic complexities.

Other important observations appear when reorganizing these data by environments to compare complexity types (Figure 2). In all environments, the variable eliciting the most amount of information on average is luminance. Spatial information comes second, and chromatic information comes third. The only exception is snowy rural environments where the mean amounts of spatial information are even lower than those yielded by color. Another key observation from Figure 2 and Figure 3 is that the distributions of complexities are different across environments and complexity types in terms of magnitudes, spread, overlap, and order of peak complexities. For example, the overlap between the distributions of spatial and chromatic complexity is larger in streets than in other environments. And the spread of the distribution of spatial complexities is smaller in malls.

### 4.2. A Model for the Distributions of Complexities

Next, we searched for a good likelihood model of how the brain processes amounts of information across environments. We hoped that the model would be analytic and parametrically simple, that is, have no more than three parameters. Furthermore, these parameters should be enough to fit the distributions, how they change across environments, and types of complexity (Figure 3). From the analyses in the last subsection and in Section 2, this model also had to have the following properties:

- Continuous Probability Density Distribution. Figure 2 and Figure 3.
- Finite Support. Complexities are bound between 0 and 1 (Equations (1)–(3)).
- Unimodal with a Peak Neither at 0 nor at 1.
- Skewed. The skewness is such that when the median > 0.5, the skewness is negative and vice versa.
- Leptokurtic

**Figure 4:**
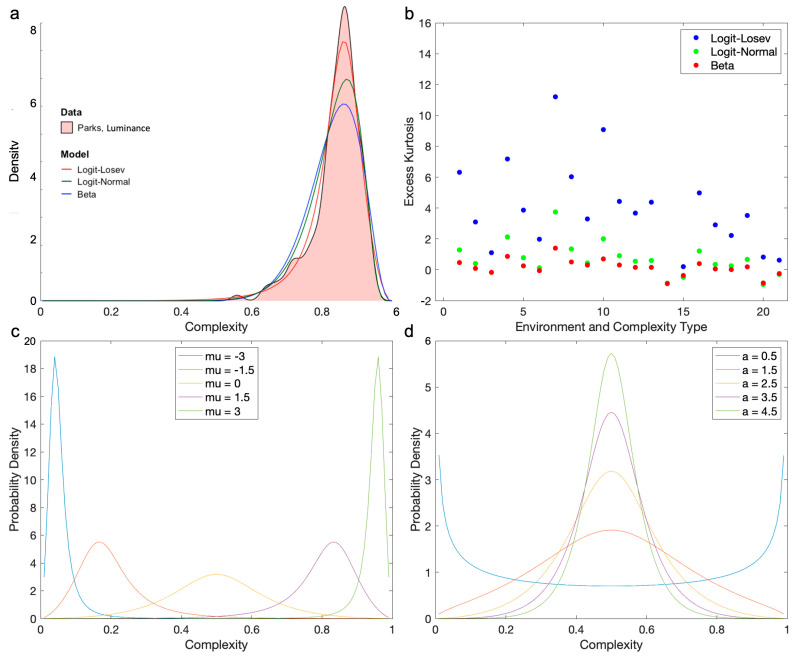
(**a**) Probability density distribution of luminance complexities in the park environment with best fits by the Logit-Losev, Logit-Normal, and Beta models. (**b**) Excess kurtoses for these three models. This horizontal axis is organized by triplets of complexity type (luminance, spatial, and chromatic in order) in the seven environments. The environments in order are parks and lakefronts, college campuses, small streets, large streets, snowy rural settings, malls, and forests. Thus, the twenty-one abscissas of Panel (**b**) are parks/luminance, parks/spatial, parks/chromatic, campus/luminance … forest/chromatic. Taken together, Panels A and B show that the Logit-Losev distribution gives a better fit because of its large positive excess kurtosis, implying more curvature at the peak. (**c**) Logit-Losev curves parametric on *µ* with *a =* 2.5. (**d**) Logit-Losev curves parametric on *a* with *µ =* 0.

We searched for this model broadly, considering mathematically or computationally tens of different continuous, finite-support probability distributions [45,46]. We even allowed modifications of infinite-support distributions to make their support finite, such as by using the logit transformation [57,58]. Almost all the distributions studied did not fulfill all the requirements above. For example, some were multimodal or had peaks at 0 or 1 (for example, the Arcsine, U-quadratic, and Continuous Bernoulli distributions). Others did not show skewness or had the wrong one (for example, the Irwin–Hall, Bates, and Marchenko–Pastur distributions). However, others did not have enough positive excess kurtosis (for example, the Beta, Logit-normal, and Kumaraswamy distributions). And some were not parametrically simple (for example, the Logit-metalog). Among these distributions, the ones that came closest but did not fully succeed were the Beta and Logit-Normal distributions (Equations (4) and (5)). They had almost all the right properties, but because of their insufficient leptokurtosis, the peak was not tall enough to capture the observed distributions (Figure 4). The Beta distribution failed statistically to fit the distributions in 7 out of 21 cases. The Logit-Normal distribution failed in five cases.

The only distribution that had the right properties and fit the data well was what we called the “Logit-Losev” distribution (Equation (6)—Figure 4a). This was a modification of a distribution studied by Losev [47], itself a generalization of the hyperbolic-secant distribution. The modification was the transformation of the independent variable with the logit function, making the outcome a finite-support distribution. This distribution was introduced in this article for the first time. The general Logit-Losev distribution had three parameters. However, in this article, we used a simplified version with only two parameters by making *a = b* (Equation (6)). The fits of this version of the model were excellent (Table 1, Columns 6 and 7). Its success stemmed in part from the Logit-Losev but not the other distributions, having a high positive excess kurtosis (Figure 4b). Another reason for the success of the model was its ability to change skewness from positive to negative as the optimal complexity crossed 0.5 as *µ* varied (Figure 4c). In turn, the width and thus the amplitude of the distribution position were mostly controlled by *a* (Figure 4d). When *a* became too large, the distribution turned bimodal.

### 4.3. Fisher Information

**Figure 5:**
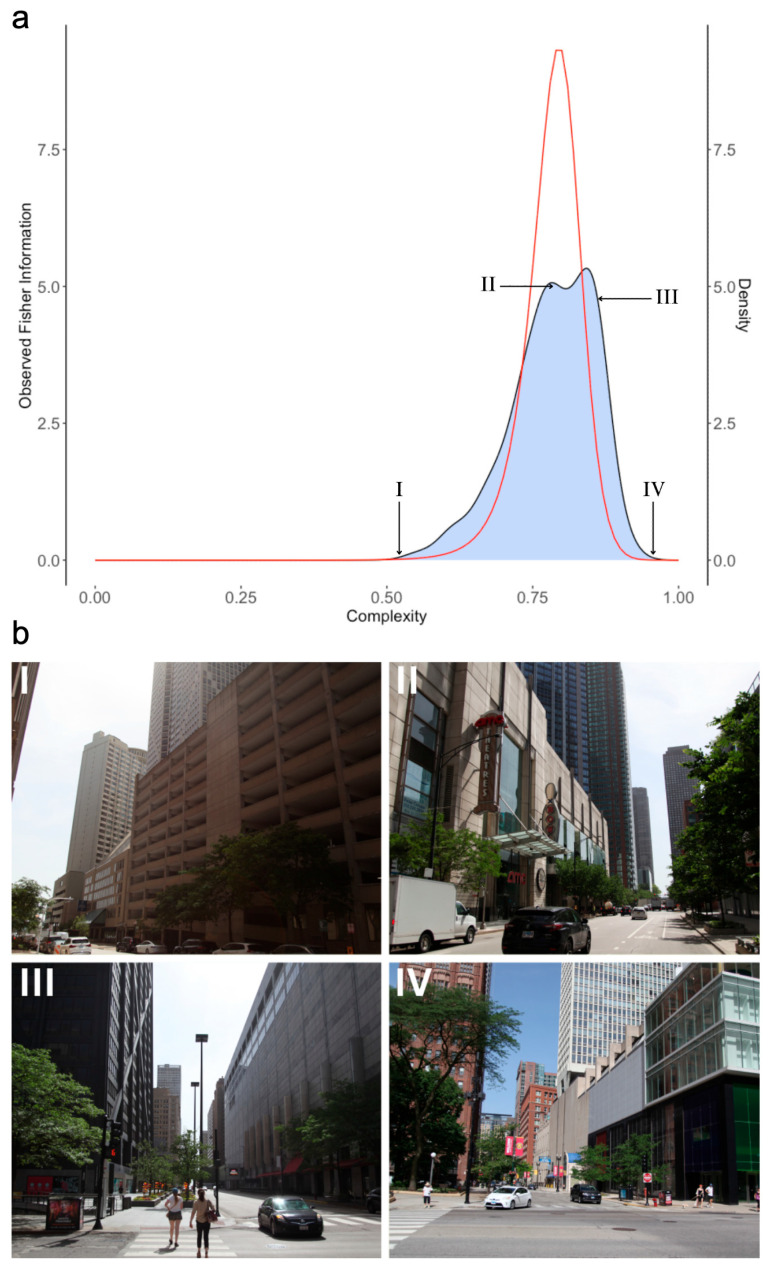
(**a**) Kernel-density distribution (shaded curve) for chromatic complexities in large streets and the corresponding Observed Fisher Information Curve (red) for the optimal Logit-Losev distribution. The curve is for the μ component of the Observed Fisher Information matrix (Equation (7)). (**b**) Four examples of images (**I**–**IV**) with complexities as indicated in A. For this environment, the peak Fisher Information is at a different complexity (**II**) than that yielding most images (**III**).

We hypothesized that the Observed Fisher Information obtained from the likelihood distribution is a measure of the aesthetic appraisal for the amount of information. Consequently, if the Logit-Losev distribution was applicable for appraisals of complexities, its Observed Fisher Information had to capture their inverted-U-shape behavior. If so, we also wanted to know what was special about the Logit-Losev distribution that allowed it to have this property. Fisher Information for the Logit-Losev distribution is a 2×2 a matrix (Equation (7)). However, not all components of this matrix are equally important. In this article, we focus on the μ-diagonal component because this parameter has a coefficient of variation 2.4 times larger than that of a (the ratio between coefficients from Columns 3 and 2 of Table 1). Thus, a can effectively be considered constant for each environment and type of complexity (but not across them). Figure 5 shows the μ-diagonal of the Observed Fisher Information for the Logit-Losev distribution for one illustrative environment. Summary data across environments appear in Table 1.

The most important observation from Figure 5a was arguably that the Observed Fisher Information curve obtained from the Logit-Losev distribution obeyed an inverted-U-shape behavior (Equation (10)). This behavior was consistent with the main hypothesis of this article. We hypothesized that the aesthetic appraisal based on the complexity of an image was due to how much it informed on the parameters of the brain model of complexities. However, four other important conclusions would follow if we accepted that the human inverted-U-shape behavior followed the Fisher Information: First, the peak of the distribution of complexities (Figure 5b(III); Complexity = 0.85) did not match that of the Observed Fisher Information (Figure 5b(II); Complexity = 0.81). Instead, the peak Observed Fisher Information complexities in Table 1 matched the medians of the distributions of complexity (supplementary information, https://osf.io/23auc (accessed on 14 November 2023)). Therefore, because of the typical negative skewness of the complexity distributions, peak Observed Fisher Information complexities were lower than the most common ones. Thus, the peak complexity might not always be the most liked [25]. Second, the Observed Fisher Information curve quantifies when the complexity is too little or too much. To illustrate this point, in Figure 5a, we took the 10%-of-peak to be our threshold of likeability. Thus, people would dislike images with complexities below 0.698 and above 0.872. Third, this range of complexities and thus the details of the inverted-U-shape curve would depend on the environment (Table 1, Column 8). Fourth, different environments would yield different amounts of maximal positive appraisal (Table 1, Column 9).

**Figure 6:**
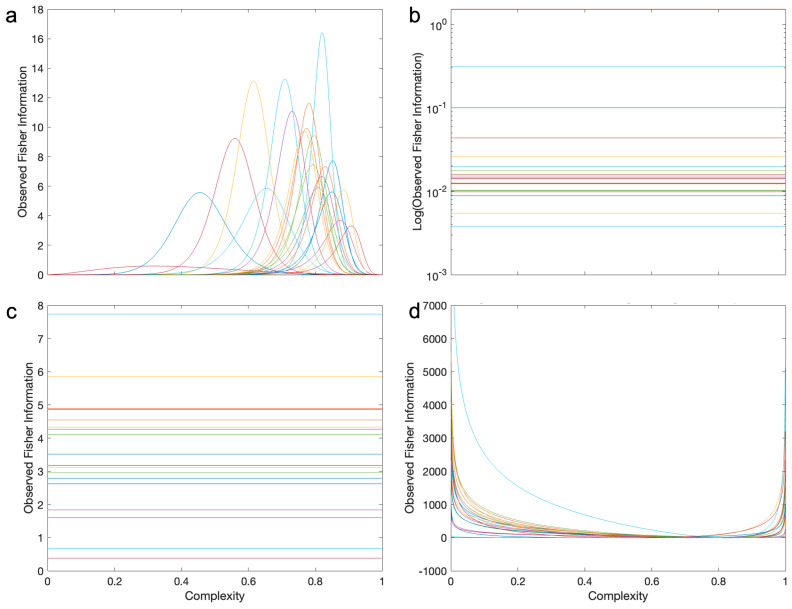
Diagonal-component curves of the Observed Fisher Information matrices for the Logit-Losev (**a**), Beta (**b**), and Logit-Normal (**c**,**d**). Distributions with optimal parameters for each environment and type of complexity. For the Logit-Losev distribution, we only show the µ component because it is what varies the most across environments. For the Beta distribution, the results for the α and β components were identical, and thus, we only show Observed Fisher Information for the former. Only the Logit-Losev distribution produces the inverted-U-Shape behavior.

The Logit-Losev model gave a better fit to the distribution of complexities than the other models that we tried and produced an inverted-U-shape behavior of the Observed-Fisher-Information curve (for example, Figure 4a—Equation (10)). However, could the other models give rise to the inverted-U-shape behavior? Figure 6 gives an answer for the models that came closest in terms of our fits. These were the alternate models in Figure 4, namely, the Beta and Logit-Normal distributions.

Even the models that came closest to the Logit-Losev distribution in terms of quality of fit could not produce the inverted-U-shape behavior (Figure 6). Figure 5 already showed that this behavior was compatible with this distribution for large streets and the µ component of the Observed Fisher Information matrix (Equation (7)). We extended this result to all environments and types of complexity (Figure 6a). In all these Logit-Losev results, we saw the inverted-U-shape behavior. The optimal complexity of this behavior is shown in mathematical form in Equation (11). Interestingly, this optimum depended only on parameter μ. In turn, the largest Observed Fisher Information in the behavior was governed by the parameter a alone (Equation (12)). However, the inverted-U-shape behavior did not apply to the other distributions. For the Beta distribution, we found that the Observed Fisher Information was independent of complexity (Figure 6b—Equation (8)). In turn, for the Logit-Normal distribution, the Observed Fisher Information was independent of complexity for the µ component (Figure 6c—Equation (9)) but exhibited a non-inverted-U-shape behavior for the µ component (Figure 6d).

### 4.4. Comparing Environments and Types of Complexity

**Figure 7:**
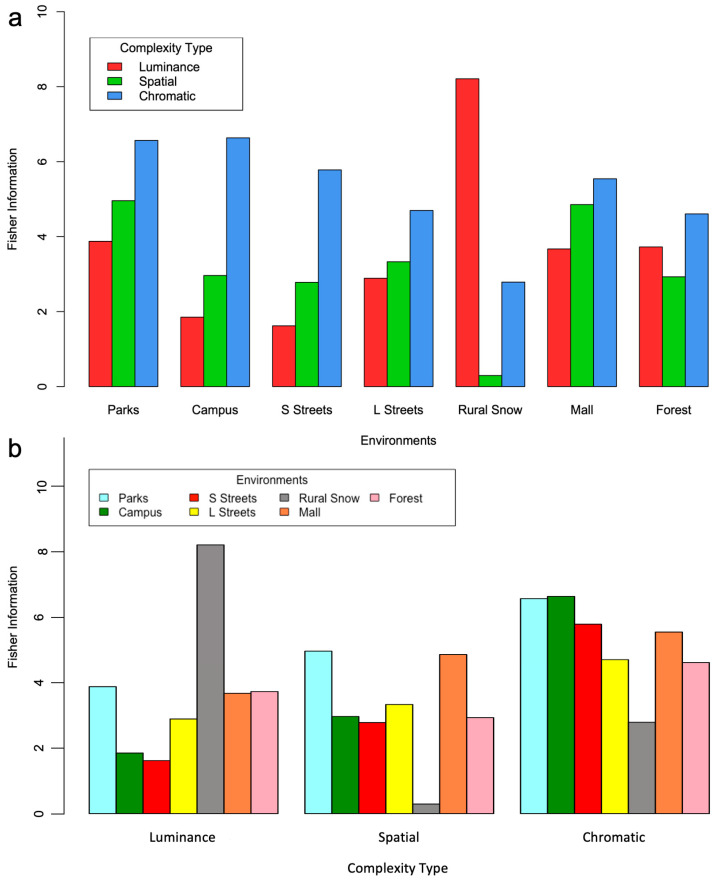
(**a**) Fisher Information as a function of environment parametric on complexity type (color-coded). (**b**) Fisher Information as a function of complexity type parametric on environment (color-coded). Chromatic and luminance complexities tend to exhibit the most and the least Fisher Information respectively. In contrast, we have observed no systematic dependence of Fisher Information as a function of environment.

Lastly, we compared the Fisher Information yielded by the distributions obtained from our seven environments and three complexity types. The goal of the comparison was to see if we could discern patterns across these conditions. Thus, for each of them, we calculated the expectation of the Observed Fisher Information, namely, the full Fisher Information. In Figure 7a, we group the expectations by environment parametric on complexity type. In turn, in Figure 7b, we group the expectations by complexity type parametric on environment.

We saw no clear systematic trends in environments and complexity types in Figure 7. Hence, neither a single environment nor a complexity type was more informative than the others overall. However, trends could be gleaned. Overall, luminance complexities yielded less Fisher Information than the others, ending in last place in five of the seven environments investigated (Figure 7a). In contrast, spatial and chromatic complexities were more informative, with the latter being especially relevant for open urban settings. The only outlier was the snowy rural environment for which only luminance complexity was helpful. In terms of environments, two appeared especially informative in comparison to the others across complexity types, namely, parks and malls (Figure 7b). Other environments tended to be less informative for some complexity types but not others (for example, snowy rural settings, and small and large streets).

## 5. Discussion

Multiple experiments have shown that the aesthetic preference for sensory inputs has an inverted U-shape dependence on their complexities, that is, their amounts of information. Why does the brain relate to the amount of information in this manner? We have previously argued against the hypothesis that people like stimuli with the most common amounts of information in the surrounding environment [25]. Instead, we hypothesize that the brain likes sensory inputs that are illuminating about the parameters of its likelihood model of amounts of information in the environment. This hypothesis anchored on the likelihood distribution follows the Bayesian strategy for the brain [59]. Knowing the right likelihood parameters may help the brain allocate the right volume of resources for future inputs [4,19,20,21]. The process involved in preparing this allocation is similar to the adaptation of likelihood parameters proposed by Brielmann and Dayan [22]. Such an adaptation is also consistent with Friston’s free energy principle [60,61,62]. It proposes that the brain lessens uncertainty through predictions made by internal models that improve over time by using new sensory signals. The fastest way to find out what these parameters are is to measure the Observed Fisher Information of the sensory input. We thus propose that Observed Fisher Information captures the expected informational utility of the stimulus (Figure 1A). Thus, too little or too much Observed Fisher Information would tend to be disliked. Therefore, we show that likelihood models that capture the distribution of the amount of information across environments lead to inverted-U-shape Observed-Fisher-Information functions.

To summarize these points, our main innovative contributions to existing computational theories of aesthetic preference are as follows: (1) We propose the existence of brain circuitry to estimate the distribution of the amounts of sensory-signal information in the current environment. (2) This estimate helps the brain allocate the right volume of resources to process future sensory signals. (3) The estimation uses a likelihood distribution of complexities that captures their probabilities the real world. (4) Upon the arrival of a sensory signal, its goodness in helping estimate the parameters of the likelihood distribution is obtained through the Observed Fisher Information. (5)The more Observed Fisher Information the signal has, the more aesthetic pleasure will arise.

### 5.1. Limitations

Before continuing with a discussion of these findings on complexities across environments, we address four limitations of our study. First, a limitation is that we only sampled locations in the region around our university. One can imagine other locations around the world whose Observed Fisher Information would not have an Inverted U-shape dependence on complexities. However, we feel confident that our locations are a broad first set. They cover exterior and interior urban images (such as streets and a mall) and natural ones (such as a forest and snowy fields). Second, the inverted-U-shape relationship for complexity and preference is not necessarily a given and could show individual differences [63]. Our study focuses on the statistics of the environment and thus, does not address individuality. However, individuals learn parameters of the environment differently [29,30], and thus, an extension of the model could capture individuality. Third, the current data may not tell us something categorical about systematic differences between environments. We only sampled one location per type of environment, for example, only one forest. Other forests may behave differently than ours. Still, if they do, we believe that we should not group them in a single environment. Fourth, the data in this article only allow us to build the theory for the visual system. Nevertheless, that we show an inverted-U-shape behavior with sound complexities [64,65] suggests that our theory may extend to the auditory system too.

### 5.2. A Likelihood Function Fitting the Distribution of Complexities

Our efforts led us to search for and find a likelihood function model that could fit the distribution of complexities across different environments well. The model that we found was the Logit-Losev function. The process of finding this function taught us important lessons about the best likelihood model for the brain. Most simple, standard, analytic models that one finds in the literature cannot fit the distribution of complexities of real-world environments. Good models must be continuous, smooth, unimodal, with finite support, and with large skewness and positive excess kurtosis. Not only that, but the sign of the skewness must vary systematically with the peak of the distribution. The skewness is especially important because it causes the median of the distribution to be separate from its mean and peak. As we report, the complexities yielding the most Observed Fisher Information when using the Logit-Losev distribution are close to the median, not the other statistics. However, one cannot generalize and conclude that our hypothesis predicts that the preferred level of complexity is always near the median of the distribution. Our search space includes many distributions, converging to Logit-Losev, but with broader explorations, we may find other ones. Any of them should have the intricate properties of the Logit-Losev in terms of, say, kurtosis and skewness. However, the Observed Fisher Information depends on the parametric structure of the distribution. Consequently, we cannot guarantee that its median would be close to the optimal complexity.

But is the fit of the Logit-Losev function too good? This concern arises because χ^2^ of the fit is lower than the degrees of freedom in many entries of Table 1. When the ratio between these two quantities, the so-called reduced-χ^2^ statistic, is smaller than 1, we may have overfitting [66]. Consequently, our model could correspond too closely to our data and may thus fail to fit more observations reliably. However, we reject overfitting for our results based on three arguments: First, our model has only two parameters to fit a complex family of curves. Second, Table 1 is already a test of whether the model does not fit more observations reliably after producing a fit with reduced-χ^2^ statistic smaller than 1. For example, although such a result is seen for Parks with luminance complexity, a good fit is also seen in other combinations of environments and complexities. Third, our model has a nonlinear dependence on the parameters. However, one can only reject fits based on reduced-χ^2^ statistics for linear models, that is, those that are linear superpositions of basis functions [67]. We conclude that the Logit-Losev function does not overfit the distributions of complexity across environments.

Distributions that, like the Logit-Losev function, have all these properties can be “blueprints” for calculating the amount of Observed Fisher Information for each environment and complexity type. Such functions may thus allow us to differentiate useful from useless information. Too little Fisher Information for low-complexity sensory inputs is equivalent to being useless by being “boring” (Figure 1D). Too little Fisher Information for high-complexity stimuli is equivalent to being useless by being “confusing” (Figure 1D).

### 5.3. Variation in the Distribution of Complexities

An important pattern appearing in our data is that different environments tend to vary systematically in terms of their distributions of complexities. The most obvious example is the snowy rural environment, whose complexities peak near zero. The main reason for a near-zero peak is the high albedo of snow, which causes substantial amounts of light to reflect at all wavelengths. Such a near-zero peak causes the unique negative excess kurtosis of this distribution. Moreover, this environment also shows the most spread of density distributions of spatial and chromatic complexities. One reason for this spread is that the snowy rural environment occasionally has woodlands, whose higher complexities match those of forests. In contrast to snowy rural environments, streets have the highest complexities, while forests and parks tend to have among the lowest values, albeit still above those of snow. Forests have low complexities because they tend to be monochromatic, isoluminant, and spatially simple. Curiously, parks, which are a result of cultural evolution [68,69,70], tend to have some of the same properties of forests and thus, relatively low complexities. Again, this is not surprising because parks are healthy, urban green spaces [71,72,73], often reminiscent of forests.

### 5.4. Different Types of Complexities

Theoretically, different complexity types are possible. However, do humans perceive distinct types of complexities or a single, composite one for the whole image? If the latter possibility is correct, then how does the brain combine the different complexity types that give rise to perception? Many possibilities exist, and of course, one can test them experimentally. The simplest of these possibilities is a weighted computation of the complexity types. One way to develop these weights would be to consider that, typically, luminance complexity is higher than spatial complexity, which is higher than chromatic complexity. Alternatively, one can weight according to Fisher Information. Thus, the opposite ranking is true, with chromatic Fisher Information being typically the highest and luminance Fisher Information being the lowest. A much more computationally complex but mathematically sensible alternative to weight ranking would be to compute a single complexity index. The brain could do this by combining the various variables (for example, luminance, space, color, and others) into a single multidimensional one. The disadvantage of such a combination is that it suffers from the curse of dimensionality [74,75].

Relatedly, the different complexity types could interact with other aesthetic variables. In this article, we followed several other studies [5,6,7,15,24,44,76,77] and studied luminance, spatial, and chromatic complexities in isolation. However, recent studies have looked at how complexity competes with other variables to find whether an image is pleasant [25,29,78,79]. For example, an image with a lot of balance and symmetry is less complex. However, balance and symmetry are also aesthetic variables. Hence, different people decide individually how to equilibrate complexity, on the one hand, and balance and symmetry, on the other hand, in their appraisal of images [25]. Variables like content, meaning, and significance are also part of this equilibrium [80,81,82]. Importantly, balance, symmetry, content, meaning, and significance are all sources of information, as is complexity. However, in a sense, complexity is a meta-variable, changing in response to the former sources of information. Complexity informs on an amount of information. We have suggested that the brain may measure the amount of information (including symmetry, balance, content, meaning, and significance) to allocate resources. In turn, the brain measures, for example, symmetry, to decide if one should analyze whether the object is a face, a leopard, or a fruit.

### 5.5. Adaptation to Different Environments

Because environments can be rich, our brain could use not just a single complexity likelihood function but multiple ones [4,15,44,77]. However, our findings suggest that a single, simple likelihood function is sufficient to capture each or most real-world environments. Even environments behaving differently from the rest (for example, snowy rural settings) are captured well enough. However, questions arise on how the brain sets the parameters for each environment. For example, how quickly and correctly do internal parameters adapt after environmental changes [22,29,30]? Does experience shape one’s ability to adapt the parameters to future new environments? For example, if people live their entire lives in the jungle and have an internal model fully perfected to rainforest characteristics, can they adapt efficiently when moving to a big city? If given enough time and energy, can we adapt to any environment? What if the environment had naturally or artificially created complexity distributions that did not match our internal likelihood function?

The importance of answering these questions on adaptations to new environments is that the responses have implications for how our aesthetic values change over time. Recent studies have shown that aesthetic values are constantly drifting [83]. Our results suggest that one reason for the drift is adaptation to the environment. A prediction of this adaptation is that even the inverted-U-shape curve is not immutable. Measuring this curve in two different environments could lead to distinct locations of the peaks of the inverted-U-shape even when testing on identical sensory stimuli.

### 5.6. Further Tests of Our Theory

This is a theory article, and thus, we have not conducted experiments to test whether the brain follows what we are proposing. Nevertheless, the last section proposes experimental tests of our theory related to adaptation to different environments. Furthermore, the section before that addressed predictions related to the distinct types of complexity in an image. Another set of interesting tests would arise if a person were in an environment where the complexity statistics are not captured by the Logit-Losev-like function. We do not know whether such an environment exists in the natural or urban world. However, we can create environments like this artificially. We predict that if adaptation takes place fast enough, the brain will try to find the best Logit-Losev parameters, although the fit would not be perfect. With these parameters, we can predict what images the subject would like and dislike. Without adaptation, these parameters would stay constant, but we can still make these predictions. In an even stranger environment, we can make all images have the same complexity but vary among other dimensions. For example, consider an image with only red and black pixels. Now, take a second image that is identical to the first, except that every pixel that is red in the first is now green. These two images have the same luminance, spatial, and chromatic complexities. However, some people may prefer the “red” image while others may prefer the “green” one [83]. Thus, these people would not have a preference based on complexities but based on dominant color, or other dimensions in alternate examples. In contrast, if we control these other dimensions, but allow complexity to vary, we predict that images with similar complexities will have the same preference regardless of the distribution of complexity in the artificial environment.

### 5.7. Generalizing the Use of Observed Fisher Information

In this work, we have shown the possible utility of Observed Fisher Information to account for the aesthetic appraisal of complexities. Could Fisher Information be useful with other aesthetic variables? In rough strokes, the brain interprets a sensory variable as possibly carrying aesthetic information if the signal is important for survival. If this happens, the brain likely dedicates special circuit to this signal, making its processing fluid. This fluidity has been considered as a proxy to a positive aesthetic appraisal [27,28]. If so, this proxy may be showing high Observed Fisher Information, which may help the brain set the parameters of one of its multiple internal models about the world. Thus, Fisher Information may provide a method of quantifying some theories of aesthetic emotions, such as the processing fluency theory [27,28].

## Figures and Tables

**Table 1:** Fits and Observed Fisher Information with the Logit-Losev distribution. Columns 3 and 4 are the parameters (a and µ) of the fit. Columns 5–7 are the statistical test of the null hypothesis that the fits are adequate. Column 8 is the complexity yielding the optimal Observed Fisher Information. Column 9 is the optimal Observed Fisher Information.

	Complexity Type	a	µ	DF	χ^2^	*p*-Value	Complexity of Optimal Observed Fisher Information	Optimal Observed Fisher Information
Parks	Luminance	2.78	1.75	47	33.8	0.92	0.851	7.73
	Spatial	3.15	1.23	57	42.2	0.92	0.773	9.92
	Chromatic	3.62	0.47	67	50.3	0.93	0.615	13.1
Campus	Luminance	1.92	1.94	67	74.9	0.23	0.897	6.05
	Spatial	2.43	1.44	67	68.0	0.44	0.808	5.90
	Chromatic	3.64	0.89	67	69.0	0.40	0.708	13.2
Small Streets	Luminance	1.82	2.29	57	40.5	0.95	0.908	3.31
	Spatial	2.37	1.73	67	76.0	0.21	0.849	5.62
	Chromatic	3.41	1.27	57	46.5	0.83	0.780	11.6
Large Streets	Luminance	2.40	2.04	57	46.6	0.83	0.884	5.76
	Spatial	2.58	1.51	57	46.4	0.84	0.819	6.66
	Chromatic	3.07	1.36	57	44.0	0.89	0.795	9.42
Snowy Rural	Luminance	4.05	1.52	47	27.5	0.98	0.820	16.4
	Spatial	0.768	−0.76	37	41.1	0.29	0.318	0.590
	Chromatic	2.36	−0.18	37	30.3	0.77	0.455	5.57
Malls	Luminance	2.71	1.58	57	25.7	0.99999	0.822	7.34
	Spatial	3.12	1.19	57	51.9	0.66	0.782	21.3
	Chromatic	3.33	0.998	57	40.5	0.95	0.730	11.1
Forest	Luminance	2.73	1.34	57	32.3	0.996	0.792	7.45
	Spatial	2.42	0.64	77	53.4	0.98	0.654	5.86
	Chromatic	3.04	0.24	87	52.6	0.998	0.559	9.24

## Data Availability

All data are in the Supplementary Materials reported above.

## Supplementary Materials

The following supporting information can be downloaded at: https://osf.io/23auc (accessed on 14 November 2023), Photography and Data: Pictures and Data by Environment; Statistics: Statistics Table 1.
